# Polymers in Sensor and Biosensor Design

**DOI:** 10.3390/polym13060917

**Published:** 2021-03-16

**Authors:** Almira Ramanaviciene, Ieva Plikusiene

**Affiliations:** NanoTechnas—Center of Nanotechnology and Materials Science, Faculty of Chemistry and Geosciences, Institute of Chemistry, Vilnius University, Naugarduko st. 24, LT-03225 Vilnius, Lithuania; ieva.plikusiene@chgf.vu.lt

The growing demand and need for new analytical instruments that are highly sensitive, fast, and user-friendly for detecting various analytes has opened up new possibilities and led to the development of novel sensors and biosensors. To date, there has been a high interest in the synthesis of polymers, copolymers, and nanocomposites characterized by unique properties. Contemporary evidence suggests that sensors and biosensors designed to employ different polymers exhibited higher sensitivity, lower limits of detection, wider linear detection ranges, more efficient electron transfer, and improved stability. The combination of new polymers with nanoparticles ensuring special electrical and catalytic properties promotes their successful application in sensors and biosensors [1,2].

Conjugated and conducting polymers can be synthetized chemically and electrochemically. One the most attractive method for the synthesis of π–π conjugated polymers, such as polypyrrole and polyaniline, is an enzymatic reaction-based polymerization initiated by the enzyme glucose oxidase [3]. Polymers and their composites with different gold nanoparticles were successfully synthetized and applied for the development of enzymatic biosensors with a wide linear range, improved stability, and high anti-interference ability to electrochemically active substances present in real samples [4]. Aptamer-functionalized silver nanoclusters combined with polypyrrole nanoparticles were used for the development of fluorescent aptasensor for the *Staphylococcal enterotoxin* A detection [5]. Biocompatible conjugated polymers were successfully used as a matrix for biomolecules (enzymes, antigens, antibodies, cells) entrapment or immobilization [6]. Conjugated polymers and copolymers stand out among other polymers by their environmental stability, flexibility, and attractive electrochemical and electrochromic properties [7,8,9] that can be enhanced by gold nanostructures electrochemically deposited on the electrode. In addition, conducting polymers offer a variety of technological solutions important for the development of sensors, biosensors, and biofuel cells [10,11,12].

A modern approach and a great solution for developing mediator sensors and biosensors is the application of polymers containing redox-active sites or groups that can be reversibly oxidized and reduced. The redox-active polymers are frequently used for oxidoreductase immobilization, providing better biosensor stability and reagent-free approach for analyte detection. The efficient electron transfer between the enzyme redox center and the surface of the electrode ensured by these polymers improved the operation of biosensors [13,14] and enzymatic biofuel cells [15]. Bovine serum albumin and chitosan-based biocompatible redox-active polymers with covalently bound redox mediators and containing carbon nanotubes have successfully been used for bacteria immobilization and for biochemical oxygen demand concentration registration [16].

Extensive research has been carried out on the preparation and application of molecularly imprinted polymers (MIPs) as analyte recognition materials for chemical, biochemical, biological, and biomedical applications due to their high selectivity and affinity, long stability, resistance to pressure, high temperatures, and extreme pH. Ion-imprinted polymers were successfully applied for the determination of heavy metal ions [17]. MIP technology enables the recognition of small molecules, such as caffeine [18], theophylline [19], histamine [20], uric acid [21], and DNA [22], enzymes [23], proteins [24] or whole cells [25]. The pandemic caused by severe acute respiratory syndrome coronavirus 2 (SARS-CoV-2) has brought together researchers from around the world to develop new methods and analytical systems to diagnose COVID-19. It is a major challenge to develop robust, sensitive, specific, less expensive, portable, and well-operating sensors and biosensors for the SARS-CoV-2 virus and its spike and nucleocapsid protein detection. An article about MIP-based portable electrochemical sensors for the detection of SARS-CoV-2 nucleoprotein has just been published [26].

One of the critical problems in cancer diagnostics is the late detection of the disease. In some cases, this problem can be solved by the timely detection of cancer biomarkers present in the body fluids. For this purpose, a special type of biosensor, namely, immunosensors based on conducting polymers, were developed [27]. A new approach for cancer biomarker detection based on the conjugated polymer polypyrrole modified with antibodies was established for novel prostate-specific cancer biomarker (CCR4) determination [28]. Various label-free, sensitive, and disposable immunosensors based on different polymers were developed. Extensive research has been carried out identifying cancer biomarkers [29] and detecting cancerous and metastatic cells, successfully discriminating them from normal cells. MnO_2_-decorated polymer dots have successfully been applied for the development of a wireless label-free electrochemical sensor for cancer cell detection [30]. It was shown that MCF-7 human breast cancer cells can be detected using a nanocomposite consisting of polymer and multiwall nanotubes in a dual aptamer-based sandwich-type biosensor [31].

This Special Issue of *Polymers* entitled “Polymers in Sensor and Biosensor Design” is dedicated to the topic research articles and reviews on the application of polymers, copolymers, and nanoparticles in sensors and biosensors design. Significant attention will be paid to conducting and redox polymers that are improving charge transfer, to electrochromic polymers and their application for different purposes. Papers dedicated to the synthesis and application of MIPs for small molecules, DNA, different biomarkers, or cells are preferred. Innovative, original, and multidisciplinary studies about the development of sensors and biosensors with improved properties based on polymers, copolymers, or nanocomposites for the detection and quantification of chemical compounds, biomolecules, antigens, antibodies, viruses, and cell will be considered for publication. We also welcome research about advanced COVID-19 diagnostics.
